# Profiling of 17 Y-STR loci in Mazandaran and Gilan provinces of Iran

**DOI:** 10.3906/sag-1808-179

**Published:** 2019-10-24

**Authors:** Minoo SAYYARI, Ali SALEHZADEH, Mohamad Amin TABATABAIEFAR, Ali ABBASI

**Affiliations:** 1 Department of Biology, Rasht Branch, Islamic Azad University, Rasht Iran; 2 Department of Genetics and Molecular Biology, School of Medicine, Isfahan University of Medical Sciences, Isfahan Iran; 3 Pediatric Inherited Diseases Research Center, Research Institute for Primordial Prevention of Noncommunicable Disease,Isfahan University of Medical Sciences, Isfahan Iran; 4 Iranian Legal Medicine Research Center, Legal Medicine Organization, Tehran Iran

**Keywords:** DYS385b, DYS391, haplotype diversity, Y-chromosome

## Abstract

**Background/aim:**

The Y-chromosome mainly consists of heterochromatin regions that have a father-to-son inheritance. Short tandem repeat polymorphic (STRP) markers distributed all over the chromosome provide the opportunity for investigations in forensic medicine and ancestral lineage studies. Due to the existence of wide varieties of geographical and ethnic groups in Iran, studying Y-STRP markers is necessary for further applications. Here we investigated the provinces of Mazandaran and Gilan for the first time.

**Materials and methods:**

Samples included 119 and 90 unrelated males from Mazandaran and Gilan, respectively. Using a PCR amplification kit, 17 Y-STRP markers were amplified and genotyping was conducted by capillary electrophoresis. Allele frequency, haplotype diversity (HD), and haplotype discrimination capacity (DC) were calculated. The populations were compared together and to neighboring countries including Afghanistan and Azerbaijan by F_ST_ index.

**Results:**

A total of 204 unique haplotypes were observed. No uniqueness was observed between the two provinces. HD was 0.9993 and 0.9998 in Mazandaran and Gilan, respectively. DC was 0.9666 and 0.9888 for Mazandaran and Gilan, respectively. DYS385b and DYS391 had the most and least polymorphic content in both provinces, respectively. There was not a significant difference between these two provinces (F_ST_ = 0.0006 and P = 0.00) and neighboring countries.

**Conclusion:**

The results highlight the effectiveness of these Y-STRP markers for male discrimination in the north of Iran. Using additional markers along with extended sample size would provide a better opportunity for removing matched haplotypes and introducing the best polymorphic markers in this specific population.

## 1. Introduction 

The Y-chromosome is highly heterochromatic consisting of repetitive DNA sequences, which makes sequencing difficult (1). It mainly carries genes involved in sex determination and spermatogenesis (2). During meiosis small regions at extreme ends of short and long arms of the Y chromosome, PARI and PARII, undergo recombination while other parts which contain about 95% of the Y-chromosome tend to be transmitted from father to son intact (3). This phenomenon makes the Y-chromosome interesting for studying male identification.

Short tandem repeat polymorphic (STRP) markers are a class of multiallele sequences distributed frequently and randomly all over the genomes of animals and plants. They normally consist of 1–6 bp tandemly repeated units. STRP markers are variable in length among individuals except for monozygotic twins. They are mostly located in noncoding regions of the genome (4,5). The STRP markers have been widely used in medical genetics for tracking disease genes in Mendelian inherited disorders in families but not in mutation identification (6). When several STRP markers are used simultaneously following haplotype reconstruction, they have the ability to identify individuals. Then they are commonly utilized in forensic medicine for paternity testing, identification in sexual abuse cases, criminal subjects, and proving male kinship with the survivors in mass disasters (7,8). Furthermore these markers are useful for investigating migration and common ancestral lineage of a specific population (9).

 More than 3000 Y-STRP markers have been found up to date (10), which raises the need of finding informative markers in specific populations for forensic applications, and finding ancestral lineages. Iran, a large country with more than 80 million people, consists of several ethnic groups, and needs its own ethnic-specific databases for allele frequencies and heterogeneity of STRP markers. Although the eastern and central regions have been studied previously (11–14), there is not much information on Y-STRP markers and haplotype heterogeneity in the north of Iran. Therefore, in this project, we analyzed 17 Y-STRP markers in male populations in two northern populations of Iran via multiplex PCR and capillary electrophoresis following statistical analysis.

## 2. Materials and methods

The present investigation was approved by review boards and the ethics committee of Islamic Azad University of Rasht and the Iranian Legal Medicine Research Center. Interviewing the male clients and volunteers, blood samples from 119 unrelated subjects from Mazandaran Province and 90 unrelated cases from Guilan Province were obtained on Whatman FTA Classic cards after obtaining informed written consent. Subjects enrolled in the study included males who did not belong to Sadat ancestry and did not experience blood transfusion in the last 24 h. A punch of 1.2 mm^2^ was used for DNA extraction according to the manufacturer’s standard procedure.

Using the AmpFLSTR Yfiler PCR amplification kit (Applied Biosystems, Foster City, CA, USA) 17 STRP markers were amplified in a multiplex PCR reaction following the manufacturer’s instruction. Characteristics of the markers are shown in Table 1. The PCR products were separated using capillary electrophoresis on an ABI 3500 genetic analyzer (Applied Biosystems, Foster City, CA, USA). The alleles were assigned by GeneMapper ID-X v.1.3 software (Applied Biosystems, Foster City, CA, USA). Alleles have been named following the update of the recommendation of the DNA Commission of the International Society of Forensic Genetics (15). Allele frequency for each locus and haplotype frequency were calculated by simple gene counting method. Gene and haplotype diversity (HD) was calculated according to Nei’s formula (h=N/(N-(1-ΣP_i_^2^))) using the Arlequin Software package v3.5.2.2 and GenAlEx 6.5 software. Haplotype match probability (HMP) was calculated according to HMP = 1–HD formula. Haplotype discrimination capacity (DC) was calculated by dividing the total number of unique haplotypes in the analyzed population by the total number of individuals in the sample size. Genetic distance between these two provinces and neighboring countries, Afghanistan and Azerbaijan, were calculated via FST value.

**Table 1:** Characteristic of Y-STRP markers used in this investigation.

marker STRP	Repeat motif	Allele range
DYS456	AGAT	13–18
DYS389 I	(TCTG)_3_(TCTA)n	9–17
DYS390	(TCTA)2(TCTG)n(TCTA)n(TCTG)nTCA(TCTA)2	17–28
DYS389 II	(TCTG)n(TCTA)Nn28(TCTG)2(TCTA)n	23–34
DYS458	GAAA	14–20
DYS19 / DYS394	(TAGA)_3_TAGG(TAGA)n	10–19
DYS385a/b	GAAA	7–25
DYS393	AGAT	8–16
DYS391	(TCTG)3(TCTG)n	6–14
DYS439	AGAT	8–15
DYS635	TSTA	20–26
DYS392	(TAT)n	7–18
Y GATA H_4_	TAGA	8–13
DYS437	TCTR	13–17
DYS438	TTTTC	8–13
DYS448	AGAGAT	17–27

## 3. Results

In this investigation, the genotype of 17 STRP markers was determined by the multiplex PCR followed by capillary electrophoresis in 119 males from Mazandaran and 90 males from Gilan. All loci were successfully amplified in both populations. Observations displayed a total of 204 unique haplotypes in both provinces. We found 115 unique haplotypes in Mazandaran, none of which matched the haplotypes found in Gilan (Table 2). Haplotype reconstruction unraveled 89 unique haplotypes in Mazandaran without any similarity to those seen in Gilan (Table 3). Unique haplotypes had frequencies of 0.0083 and 0.0111 in Mazandaran and Gilan, respectively. Haplotypes were submitted to the Y-Chromosome Haplotype Reference Database (YHRD: www.yhrd.org) and are available with the accession number YA004258 for Mazandaran and YA004257 Gilan. HD was 0.9993 (HMP = 0.0007) and 0.9998 (HMP = 0.0002) in Mazandaran and Gilan, respectively. We obtained DC = 0.9663 in Mazandaran and DC = 0.9888 in Gilan. The genetic distance between these two provinces was 0.003. There was a significant difference between Mazandaran and Gilan (FST = 0.0006, P = 0.00). We compared Mazandaran Province with available data from neighboring countries, Afghanistan and Azerbaijan, the statistical analysis of which revealed FST = 0.003, P = 0.00 and FST = 0.0086, P = 0.00, respectively. These statistics were FST = 0.0026, P = 0.00 and FST = 0.0082, P = 0.00 for Gilan Province in comparison with Afghanistan and Azerbaijan, respectively. The STRP markers showed a genetic diversity ranging from 0.480 to 0.863 in Mazandaran. Genetic diversity ranged from 0.379 to 0.824 in Gilan. DYS389b had the most polymorphic content in both provinces while DYS391 was the least polymorphic STRP marker (Table 4). Allele frequency per marker in both populations is exhibited in Table 5. The mean gene diversity in Mazandaran and Gilan was 0.665 and 0.683, respectively. 

## 4. Discussion

Studying Y-STRP markers in specific populations and geographical regions is valuable for identifying ancestral lineage and forensic applications. In the present study, we aimed to study 17 Y-STRP markers in two northern provinces of Iran, Mazandaran and Gilan, using STRP markers. Here we found 204 unique haplotypes and introduced DYS385b as the most polymorphic marker in both populations. No shared haplotype was observed between these two provinces. DYS391 had the least polymorphic content in both populations. Comparing these two populations together and with neighboring countries showed low genetic differences. 

 Amplification of all 17 Y-STRP markers signifies the efficiency of these markers for forensic and molecular investigation of the Y-chromosome in the north of Iran. Haplotype analysis via 17 Y-STRP markers of 259 males from eastern provinces of Iran showed HD = 0.9999 (12). This value was declared to be 0.997 in Tehran and Isfahan provinces by analyzing 9 Y-STRP markers (11). By analyzing 8 Y-STRP markers in 103 unrelated males from Isfahan Province, Vatandoost et al. announced that HD = 0.986 (14). Here we obtained HD = 0.9993 in Mazandaran and HD = 0.9663 in Gilan. Both provinces showed a lower HD by using these STRP markers when compared with eastern provinces of Iran, while the value is greater for Gilan in comparison with Tehran and Isfahan. This value was reported as 0.9850 in Afghanistan (16) and 0.9992 in Turkish Cypriots (17). The lower HD obtained for Mazandaran demonstrates that there has been less migration to this province; on the other hand, the male population in this province is isolated. The calculated DC in Gilan was higher than the previously reported DC in Tehran (DC = 0.9), Isfahan (DC = 0.938 and DC = 0.95) (11,14), and the eastern population of Iran (DC = 0.9884) (12), which illustrates the capability of these markers for male identity in this province. However, the DC value in Mazandaran was higher than the one in other provinces except for eastern provinces including Razavi Khorasan, South Khorasan, and Sistan and Baluchestan. Comparing Mazandaran and Gilan together and with neighboring countries, Afghanistan and Azerbaijan, could not establish evidence of a common ancestral lineage. It demonstrates the unique diversity among Iranian population, which is due to geographical verities and ethnic differences. 

 Our results elucidate that using Y-STRP markers has the capability of discriminating male identity among northern Iranian male subjects. Studying additional Y-STRP markers with greater sample size would help to remove matched haplotypes and determine valuable polymorphic markers in this region. This would provide a better circumstance in order to utilize ethnic-specific markers for forensic applications.
